# Eicosapentaenoic acid (EPA) efficacy for colorectal aberrant crypt foci (ACF): a double-blind randomized controlled trial

**DOI:** 10.1186/1471-2407-12-413

**Published:** 2012-09-19

**Authors:** Takuma Higurashi, Kunihiro Hosono, Hiroki Endo, Hirokazu Takahashi, Hiroshi Iida, Takashi Uchiyama, Akiko Ezuka, Shiori Uchiyama, Eiji Yamada, Hidenori Ohkubo, Eiji Sakai, Shin Maeda, Satoshi Morita, Yutaka Natsumeda, Hajime Nagase, Atsushi Nakajima

**Affiliations:** 1 Division of Gastroenterology, Yokohama City University School of Medicine, 3-9 Fuku-ura, Kanazawa-ku, Yokohama, 236-0004, Japan; 2 Department of Gastroenterology, Chigasaki Municipal Hospital, Kanagawa, Japan; 3 Department of Gastroenterology, Yokohama Rosai Hospital, Yokohama, Japan; 4 Department of Biostatistics and Epidemiology, Yokohama City University School of Medicine, Yokohama, Japan; 5 Department of molecular pharmacology and neurobiology, Yokohama City University School of Medicine, Yokohama, Japan

## Abstract

**Background:**

Colorectal cancer (CRC) is one of the most commonly occurring neoplasms and a leading cause of cancer death worldwide, and new preventive strategies are needed to lower the burden of this disease. Eicosapentaenoic acid (EPA), the omega-3 polyunsaturated fatty acid that is widely used in the treatment of hyperlipidemia and prevention of cardiovascular disease, has recently been suggested to have a suppressive effect on tumorigenesis and cancer cell growth. In CRC chemoprevention trials, in general, the incidence of polyps or of the cancer itself is set as the study endpoint. Although the incidence rate of CRC would be the most reliable endpoint, use of this endpoint would be unsuitable for chemoprevention trials, because of the relatively low occurrence rate of CRC in the general population and the long-term observation period that it would necessitate. Moreover, there is an ethical problem in conducting long-term trials to determine whether a test drug might be effective or harmful. Aberrant crypt foci (ACF), defined as lesions containing crypts that are larger in diameter and stain more darkly with methylene blue than normal crypts, are considered as a reliable surrogate biomarker of CRC. Thus, we devised a prospective randomized controlled trial as a preliminary study prior to a CRC chemoprevention trial to evaluate the chemopreventive effect of EPA against colorectal ACF formation and the safety of this drug, in patients scheduled for polypectomy.

**Methods:**

This study is a multicenter, double-blind, placebo-controlled, randomized controlled trial to be conducted in patients with both colorectal ACF and colorectal polyps scheduled for polypectomy. Eligible patients shall be recruited for the study and the number of ACF in the rectum counted at the baseline colonoscopy. Then, the participants shall be allocated randomly to either one of two groups, the EPA group and the placebo group. Patients in the EPA group shall receive oral 900-mg EPA capsules thrice daily (total daily dose, 2.7 g per day), and those in the placebo group shall receive oral placebo capsules thrice daily. After one month’s treatment with EPA/placebo, colonoscopic examination and polypectomy will be performed to evaluate the formation of ACF, and the cell-proliferative activity and cell-apoptotic activity in normal colorectal mucosa and colorectal polyps.

**Discussion:**

This is the first study proposed to explore the effect of EPA against colorectal ACF formation in humans.

This trial has been registered in the University hospital Medical Information Network (UMIN) Clinical Trials Registry as UMIN000008172.

## Background

Colorectal cancer (CRC) is amongst the most commonly encountered neoplasms worldwide [1], and both its prevalence and mortality have been increasing [2]. Removal of colorectal polyps has been to shown to reduce the risk of future development of colorectal cancer and advanced adenoma [3,4] and to thereby prevent colorectal cancer death [5]. On the other hand, patients with polyps (adenomas and/or hyperplastic polyps) also constitute a high-risk group for the development of metachronous colorectal polyps and/or CRC [6]. Therefore, a paradigm shift from surveillance for early detection of cancer or adenomas and polypectomy to new strategies for prevention, including chemoprevention, is needed to lower the burden of this disease. Several large epidemiologic and/or clinical studies have evaluated the possible effects of more than 200 agents, including fiber, calcium, and non-steroidal anti-inflammatory drugs (NSAIDs), including aspirin and selective cyclooxygenase-2 (COX-2) inhibitors, in protecting against CRC development [7]. Our group previously reported that sulindac, a NSAID, had the effect of suppressing the development of sporadic colorectal adenoma [8]. Until date, NSAIDs, especially COX-2 inhibitors, administered either alone or in combination with other agents, have shown the most promise for CRC risk reduction [4], although reports have revealed an increased risk of serious cardiovascular events associated with the use of COX-2 inhibitors [9,10]. In light of the adverse cardiovascular effects of COX-2 inhibitors and the lack of demonstrable efficacy of the other agents that had initially shown promise in this setting, novel drugs that would be both safe and effective for CRC prevention need to be developed. CRC is known to be associated with lifestyle-related diseases, such as hyperlipidemia, diabetes mellitus and obesity [11-14], therefore, we considered that these conditions might represent potential new targets for CRC chemoprevention.

Eicosapentaenoic acid (EPA) is an omega-3 polyunsaturated fatty acid (PUFA) that has long been used widely for primary and also secondary prevention of cardiovascular diseases [15]. EPA impacts the biological functions of adipocytes via two distinct mechanisms; the first, via transcriptional activation of lipogenic and adipogenic genes by binding to nuclear receptors such as Peroxisome Proliferator Activator Receptors (PPARs),[16] and the second, via direct competition with arachidonic acid (AA) incorporation into membrane phospholipids and subsequent conversion to eicosanoids, including prostaglandins (PGs). [17] Recent reports have indicated a lower incidence of colon, breast and prostate cancers in many human populations, associated with a high dietary consumption of omega-3 PUFAs. Multiple reports using a variety of rodent models of early-stage colorectal carcinogenesis, including azoxymethane- and dimethylhydrazine-induced colorectal tumorigenesis (using aberrant crypt foci (ACF) or colonic tumors as the primary endpoint), as well as the Apc^Min/+^ mouse model of familial adenomatous polyposis (FAP), have demonstrated the efficacy of the free fatty acid (FFA) form of a combination of EPA plus docosahexaenoic acid (DHA) (as fish oil substituted for the base fat source in chow).[18] In humans, a phase-III randomized placebo-controlled trial of EPA-FFA 2 g daily for 6 months was performed in 55 patients with FAP undergoing sigmoidoscopic surveillance of a rectal stump after total colrectomy. [19] Patients in the EPA-FFA arm had a significantly lower (by 22.4%) number of lower rectal polyps and a 29.8% decrease in the sum of the polyp diameters in the tattooed area of the rectum as compared with the placebo group. Importantly, daily administration of EPA-FFA 2 g was safe and well-tolerated. [19] NSAIDs chemoprevention trial set in past, first conducted to FAP patients, then applied to sporadic colorectal adenoma/cancer. Thus, much evidence suggests that EPA might be a candidate agent for CRC chemoprevention. In CRC chemoprevention trials, in general, the incidence of polyps or of the cancer itself is set as the study endpoint. Although the incidence rate of CRC would be the most reliable endpoint, use of this endpoint would be unsuitable for chemoprevention trials, because of the relatively low occurrence rate of CRC in the general population [20] and the long-term observation period that it would necessitate. Moreover, there is an ethical problem in conducting a long-term trial to determine whether a test drug may be effective or harmful.

Aberrant crypt foci (ACF), defined as lesions containing crypts that are larger in diameter and stain more darkly with methylene blue than normal crypts,[21-24] are considered as a reliable surrogate biomarker of CRC.[25] We previously reported the usefulness of ACF as a biological marker of CRC, [26,27] and carried out a chemoprevention trial for colorectal ACF. [28,29] Chemoprevention trials with colorectal ACF set as the primary endpoint may have some advantages. First, a long-term observation period is not needed to evaluate the drug effect. Our group reported the n.[29] Long-term trials need much effort and may expose the study participants to an increased risk of development of carcinoma. Second, ACF can be estimated quantitatively. Thus, we devised a prospective randomized controlled trial to evaluate the chemopreventive effect of EPA against the formation of colorectal ACF as a preliminary study prior to CRC chemoprevention trials.

This is the first clinical trial of EPA as a chemopreventive agent against colorectal ACF in humans.

## Methods/design

### Study design and setting

This study is designed as a multicenter, double-blind, placebo-controlled, randomized controlled trial to be performed in patients with colorectal ACF. It will be conducted at the Division of Gastroenterology, Yokohama City University Hospital, and its affiliate hospital, Chigasaki Municipal Hospital and Yokohama Rosai Hospital. The coordinating office shall be at the Yokohama City University Hospital, and the registration, randomized allocation and data collection shall be conducted at this site.

### Ethical considerations and registration

The study protocol is in compliance with the Declaration of Helsinki [30] and the Ethics Guidelines for Clinical Research published by the Ministry of Health, Labour, and Welfare, Japan [31]. We obtained approval for this study from the Ethics committee of Yokohama City University Hospital on May 10, 2012. The protocol and informed consent forms were approved by the institutional ethics committee at each of the participating institutions. This trial has been registered in the University hospital Medical Information Network (UMIN) Clinical Trials Registry as UMIN000008172. Written informed consent for participation in the study will be obtained from all the participating patients. The trial results will be reported in conformity with the Consolidated Standards of Reporting Trials (CONSORT) 2010 guidelines [32].

### Eligibility criteria

Patients with both colorectal ACF and resectable polyps will be recruited for the study.

The proposed inclusion criteria are as follows:

1) Age 40 to 80 years as on the date of informed consent.

2) Willingness to provide written informed consent.

The proposed exclusion criteria are as follows:

1) History of regular use of omega-3 PUFA supplements.

2) History of regular use (defined as at least once per week) of NSAIDs and/or aspirin.

3) History of heart failure, renal failure, liver cirrhosis or chronic hepatic failure.

4) History of familial adenomatous polyposis.

5) History of hereditary non-polyposis colorectal cancer.

6) History of inflammatory bowel disease.

7) Pregnancy or possibility of pregnancy.

8) Patients judged as being inappropriate candidates for the trial by the investigators.

### Intervention

All eligible patients will be allocated randomly to one of two groups, the EPA group and the placebo group. The endoscopists, doctors at the follow-up outpatient clinics, and patients will be blinded to the allocation. Patients in the EPA group shall receive oral 900-mg EPA capsules thrice daily (total daily dose, 2.7 g), and those in the placebo group shall receive oral placebo capsules thrice a day. At the end of 1 month of administration of EPA/placebo, polypectomy will be performed, and the changes in the number of ACF and in the mucosa will be evaluated.

### Outcome measurements

The primary endpoint shall be the change in the number of colorectal ACF after 1-months’s intervention. The endoscopic examinations and polypectomies will be performed using Olympus colonoscopes (model H260AZI). Bowel preparation prior to the colonoscopic procedures will be as described [33,34]. At the time of the first colonoscopy, the endoscope shall be inserted into the cecum, and the entire colorectum will be carefully observed as the endoscope is pulled back. If any polyps are detected, biopsy will be performed. Furthermore, colonic epithelial samples will be obtained. The number of rectal ACF will be counted with a magnifying endoscope, as described [25,33]. At the end of 1 month of administration of EPA/placebo, the same endoscopists will perform the polypectomy and counting of the ACF. All procedures will be recorded on DVD, and all the ACF will be photographed. The number of ACF in each patient will first be counted by the operators during the performance of the colonoscopy. To further ensure validity, the number of ACF will be counted again through observation of the recorded DVD by 3 blinded expert endoscopists (H.T, H.E, and E.S). If these expert endoscopists judge the colonoscopic examination as having been inadequate in any case, that case will be excluded.

The secondary outcomes shall be (1) the drug safety; adverse events (AEs) will be graded according to the National Cancer Institute Common Toxicity Criteria for Adverse Events (NCI-CTCAE), version 4.0. All study participants shall be provided with a study diary in order to record the daily dosage of the study treatment and the AEs. Patients developing grade 3 or more severe adverse events will be withdrawn from the study at that point; (2) Mucosal fatty acid analysis: Homogenization, extraction and derivatization of the rectal mucosa and polyp fatty acids (EPA, DHA, docosapentaenoic acid (DPA), AA, linolenic acid, linoleic acid, palmitic acid, stearic acid, etc.) shall be performed as described [35]. Fatty acid content shall be determined by gas chromatography–mass spectrometry and expressed as the percentage of the total fatty acid content [36,37]. (3) Effects of EPA on the cell-proliferative and apoptotic activities in the rectal epithelium and polyps: Colonic epithelial samples will be obtained from the same trial patients by biopsy at the time of the first colonoscopy and polypectomy. The cell-proliferative activity will be evaluated by staining for the proliferative cell nuclear antigen (PCNA) and estimation of the Ki-67 labeling indices, and the cell-apoptotic activity by the terminal deoxynucleotidyl transferase-mediated dUTP-biotin nick-end labeling (TUNEL) method. (4) Laboratory data (HDL cholesterol, LDL cholesterol, triglycerides, fatty acid fractions, fasting blood glucose, fasting blood insulin, HbA1c, blood urea nitrogen (BUN), creatinine); (5) physical examination findings (body weight, body mass index (BMI)). EPA is widely used as an anti-hyperlipidemic drug that improves the plasma lipid profile. The effect of EPA on the plasma lipid profile will be evaluated by comparing these parameters measured at the baseline with those measured after 1 month of treatment in the EPA group and placebo group. All participants will undergo a physical examination and laboratory tests at the time of the baseline endoscopic examination and polypectomy.

### Randomization

The investigator shall convey the patient’s details to the central registration center via fax. After an eligibility check, the patients will be randomly assigned to receive EPA or placebo at the central registration center by a computer program using a minimization method, with stratification by age, gender, BMI, and institution. Thus, the patient assignment will be concealed from the investigator. The randomization center will allocate a numbered treatment pack to each patient, which will contain all the drugs or placebos needed to complete a course of the trial treatment for that patient.

### Drug supply

Enteric-coated EPA capsules (Ethyl icosapentate granular capsule®) and the placebo capsules (capric, caprylic and lauric acid medium-chain triglycerides) will be purchased from Nipro Pharma Corporation Co., Ltd, Osaka, Japan. All trial drugs will be packaged identically and identified only by number. Subjects will be instructed to take one package of the trial drug after every meal each day. Compliance will be monitored by counting the empty drug packages returned by the patients at polypectomy.

### Sample size estimation

In the chemoprevention trial conducted in FAP patients, the NSAID sulindac and selective cyclooxygenase-2 (COX-2) inhibitor celecoxib reduced polyposis of the retained rectum after colectomy with ileorectal anastamosis (IRA). As previously noted, EPA has a suppressive effect for polyp formation and proliferation of FAP [19]. From these reports, we estimated that NSAIDs and EPA may have equivalent effect on suppression of polyp formation and proliferation.

Based on the target in the NSAIDs chemoprevention study for ACF, Takayama et al. reported that sulindac administration at 300 mg/d for 2 months to post-polypectomy patients suppressed ACF formation, decreasing the number of ACF from 7.70 ± 4.04 (baseline) to 4.00 ± 2.95 (at 2 months, *p < 0.001*) [33]. Presuming EPA and sulindac may have equivalent effect in suppressing ACF formation, to detect the reduction in the number of ACFs in the EPA group using the Mann–Whitney U test with a two-sided significance level of 5% and a power of 80%, it was estimated that a sample size of 12 patients per group would be necessary. Assuming a 10% dropout rate, we propose to recruit 15 patients per group, that is, a total of 30 patients.

### Statistical analysis

The number of ACFs in each group, the primary endpoint, will be compared between the EPA group and the placebo group by the Mann–Whitney U test. The safety, one of the secondary endpoints, will be compared by the chi-square test. The remaining results in the two groups will be compared by the Mann–Whitney U test or Student’s *t* test. A P values of < 0.05 will be regarded as indicative of significance. The analysis will be performed using SPSS, version 17.0 (SPSS Inc., Chicago, Il.).

### Trial Steering Committee and Data Monitoring Committee

The Trial Steering Committee and Data Monitoring Committee shall be located at the Department of Gastroenterology, Kanto Medical Center, NTT East. The committee shall consist of three people: Nobuyuki Matsuhashi, M.D., Toshio Fujisawa, M.D., and Jun Hamanaka, M.D. The Management Team will monitor the trial progress status and data by face-to-face and/or telephonic contact with each of the sites every month.

### Study flow

**Figure 1:**
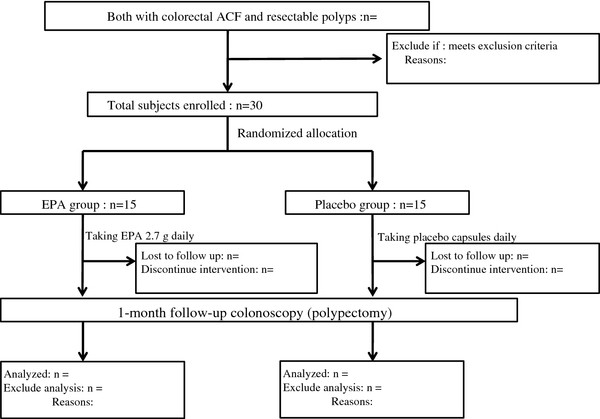
Study Flow.

A flow chart of the study is shown in Figure 1.

## Discussion

This is the first study proposed to explore the effect of EPA against colorectal ACF formation. Knowledge of the mechanisms underlying the anti-neoplastic activity of EPA remains nebulous. Current understanding of the mechanistic aspects of the anticancer activity of EPA has been reviewed in detail in published reviews [38-41]. In general, the major mechanisms proposed to underlie the anti-neoplastic activities of EPA; (1) modulation of COX activity, (2) alteration of the membrane dynamics and cell surface receptor function, and (3) increased cellular oxidative stress. However, the in vivo relevance of each of the above putative mechanisms and their relative contributions to the anticancer activity of EPA remain unclear. Several in-vitro studies have explored the anti-neoplastic activity of omega-3 PUFAs against human CRC cells, and EPA treatment has been shown to reduce cellular proliferative activity and increase cellular apoptotic activity [42-45]. EPA can act as an alternative substrate for COX-2, instead of AA, leading to a reduction in the formation of the pro-tumorigenic ‘2-series’ PGs (e.g., PGE_2_) in favor of the ‘three-series’ PGs (e.g., PGE_3_) in several cell types, including CRC cells [46-48]. Furthermore, incorporation of EPA into the cell phospholipid membrane alters the fluidity, structure, and/or function of the lipid rafts or calveolae [49], which are sphingolipid- and cholesterol-rich microdomains that float freely in the cell membrane. The localization of cell surface receptors, such as epidermal growth factor receptor (EGFR) [50], in lipid rafts is believed to be crucial for downstream receptor signaling, controlling proliferation and apoptosis [51,52]. Furthermore, EPA may exert an antineoplastic effect through alteration of the cellular redox state and of the oxidative stress exposure of the cells. PUFAs are highly peroxidizable, which generates reactive oxygen species (ROS), such as superoxide radicals. Many tumor cells display altered cellular pathways for the handling of ROS, including depletion of the major intracellular antioxidant, glutathione. Subsequent elevation of the intracellular ROS levels by EPA has been hypothesized to induce cancer cell apoptosis [53].

This trial may have the following limitations. First, ACF are considered as a reliable surrogate biomarker of CRC, [21] although their biological significance still remains controversial. In CRC chemoprevention trials, in general, the incidence of polyps or of the cancer itself is set as the study endpoint. Although the incidence rate of CRC would be the most reliable endpoint, use of this endpoint would be unsuitable for chemoprevention trials, because of the relatively low occurrence rate of CRC in the general population [18] and the long-term observation period that it would necessitate. We previously reported the usefulness of ACF as a biological marker of CRC [26,27] and carried out a chemoprevention trial for colorectal ACF [28,29]. Thus, we devised a trial using this endpoint (ACF) to evaluate the chemopreventive effect of EPA. Second, we do not propose to conduct a dose–response study in respect of the effect of EPA on ACF formation. Until now, trials of EPA for cancer prevention and adjuvant treatment have been conducted using EPA at doses of 1000 – 4000 mg per day. In Japan, the EPA drug product specification is 0.9 g, and 2.7 g of EPA has been commonly used and very well tolerated. Therefore, we planned to conduct this trial using 2.7 g of EPA per day. Third, an intervention period of 1 month may be too short to allow reliable detection of differences between the groups. However, we showed in a previous study that oral administration of metformin for 1 month suppressed the formation of colorectal ACF in humans [29]. If the intervention agents had a chemopreventive effect, an intervention period of 1 month would be sufficient to evaluate the changes in the number of ACF.

We previously conducted a short-term chemoprevention trial of metformin for colorectal ACF, and showed the suppressive effect of the drug on the formation of ACF. Thereafter, we are conducting a long-term metformin chemoprevention trial for colorectal polyps, the trial registered in the UMIN Clinical Trials Registry as UMIN000006254 [34]. We propose to repeat the same step for the chemoprevention trial using EPA.

If EPA were found to be effective for the prevention of CRC, the impact would be extremely large. We consider it of interest, therefore, to determine whether EPA might suppress the formation of human colorectal ACFs.

## Abbreviations

CRC: Colorectal cancer; NSAIDs: Nonsteroidal anti-inflammatory drugs; COX-2: Cyclooxygenase-2; ACF: Aberrant crypt foci; EPA: Eicosapentaenoic acid; PUFA: Polyunsaturated fatty acid; AA: Arachidonic acid; PG: Prostaglandin; FAP: Familial adenomatous polyposis; FFA: Free fatty acid (FFA); DHA: Docosahexaenoic acid; PCNA: Proliferative cell nuclear antigen; TUNEL: Terminal deoxynucleotidyl transferase-mediated dUTP-biotin nick-end labeling.

## Competing interests

None of the authors has any financial interests relevant to this trial to disclose.

## Authors’ contributions

TH and AN conceived the study. HT, TU and AE shall perform the baseline colonoscopy and polypectomy. HE, HO and ES will conduct another count of ACF on a DVD recording to ensure its validity. HI, SU, SM and HN shall recruit participants and follow-up at outpatient clinic. EY and KH shall carry out the pathological analyses. Analysis and interpretation of data will be conducted by YN and SM. All the authors have read the final manuscript and approve of its submission for publication.

## Current study status

This trial began recruiting patients in June 2012 and shall complete recruitment in December 2012. Data collection is due to be completed in March 2013, and the results are scheduled to be published in June 2013.

## Pre-publication history

The pre-publication history for this paper can be accessed here:

http://www.biomedcentral.com/1471-2407/12/413/prepub
